# Cell wall thickness and the molecular mechanism of heterogeneous vancomycin‐intermediate *Staphylococcus aureus*

**DOI:** 10.1111/lam.13456

**Published:** 2021-02-15

**Authors:** J. Cui, H. Zhang, Z. Mo, M. Yu, Z. Liang

**Affiliations:** ^1^ Department of Pulmonary and Critical Care Medicine Chinese PLA General Hospital Beijing China; ^2^ Department of Respiratory disease Beijing Luhe Hospital Affiliated to Capital Medical University Beijing China

**Keywords:** cell wall thickness, hVISA, molecular mechanism, MRSA, RT‐PCR, TEM

## Abstract

Methicillin‐resistant *Staphylococcus aureus* (MRSA) with reduced sensitivity to vancomycin (VAN) has caused many clinical cases of VAN treatment failure, but the molecular mechanism underlying the reduced sensitivity to VAN is still unclear. We isolated a heterogeneous VAN‐intermediate *Staphylococcus aureus* (hVISA), which was also a MRSA strain with reduced sensitivity to VAN. To investigate the molecular mechanism underlying the reduced sensitivity to VAN exhibited by the hVISA strain, we compared the hVISA strain with a VAN‐sensitive MRSA strain, known as the N315 strain. The images captured by transmission electron microscopy showed that the cell wall of the hVISA strain was significantly thicker than that of the N315 strain (36·72 ± 1·04 nm vs 28·15 ± 1·25 nm, *P* < 0·05), and the results of real‐time quantitative PCR analysis suggested that the expression levels of the cell wall thickness related genes (*glmS*, *vraR/S*, *sgtB*, *murZ* and *PBP4*) of the hVISA strain were significantly higher than those of the N315 strain (*P* < 0·05). In conclusion, this study indicated that the upregulation of the expression of the genes related to cell wall synthesis might be the molecular mechanism underlying the cell wall thickening of the hVISA strain and might be related to its resistance to VAN.

## Graphical abstract

**Significance and impact of the study:** We isolated a heterogeneous vancomycin (VAN)‐intermediate *Staphylococcus aureus* (hVISA) strain, and we demonstrated that the cell wall of the hVISA strain was significantly thicker than that of ordinary MRSA strains. The upregulated expression of *glmS*, *vraR/S*, *sgtB*, *murZ* and *PBP4* genes related to cell wall synthesis might be the molecular mechanism underlying the cell wall thickening of the hVISA strain and might be related to its reduced sensitivity to VAN. These results could be used as a supplement to other hVISA‐related studies in the world and might be beneficial to prevent and control the emergence of more hVISA.

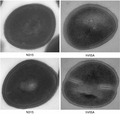

## Introduction

Vancomycin (VAN) has been increasingly used to treat various infections caused by methicillin‐resistant *Staphylococcus aureus* (MRSA). Although VAN‐resistant *Staphylococcus aureus* (VRSA) is rare, clinical cases of MRSA infection treated unsuccessfully with VAN treatment have emerged (Hiramatsu *et al*. 1997; Cong *et al*. 2020). These cases have motivated the researchers to devote more attention to *Staphylococcus aureus*, which is not yet resistant to VAN but exhibits reduced sensitivity to VAN, mainly referring to VAN‐intermediate *S. aureus* (VISA) and heterogeneous VAN‐intermediate *S. aureus* (hVISA) (Tran and Rybak 2018; Wongthong *et al*. 2020).

According to the Clinical and Laboratory Standards Institute (2006), based on the minimum MIC concentration of VAN against *S. aureus*, the susceptibility of *S. aureus* to VAN was defined as follows (Tenover and Moellering 2007): VRSA, MIC ≥ 16 *μ*g ml^−1^; VISA, MIC is between 4 and 8 *μ*g ml^−1^; hVISA, MIC ≤ 4 *μ*g ml^−1^, but its offspring contain a small number of subgroups of VAN‐mediated resistance (MIC ≥ 4 *μ*g ml^−1^); VSSA (VAN‐sensitive *S. aureus*), MIC ≤ 2 *μ*g ml^−1^. Hiramatsu *et al*. conducted pulsed field gel electrophoresis analysis on the first VISA strain (Mu50) and the first hVISA strain (Mu3 strain) and found that the two strains are closely related. And their research also found that the hVISA VAN MIC value can reach 8 *μ*g ml^−1^ from 3 *μ*g ml^−1^after screening with different concentrations of VAN, so they believe that hVISA might develop into VISA under the selective pressure of VAN and hVISA was the previous state of VISA (Hiramatsu *et al*. 1997).

Previous research showed that VISA synthesized excessive amounts of peptidoglycans rich in d‐alanyl‐d‐alanine residues, which led to an increase in free side chains and cell wall thickening (Lowy 2003). VAN bound to these free side chains cannot pass through the cell wall and exert to its antibacterial activity, which might be a mechanism underlying the reduced VAN susceptibility (Cui *et al*. 2003). However, it is unclear whether the thickening of the cell wall and the molecular mechanism were also related to hVISA and its reduced sensitivity to VAN.

Our research team collected 130 early‐stage MRSA strains in our hospital from 2013 to 2018, and no VRSA strain was identified among these 130 strains. However, one hVISA strain was isolated from the 130 MRSA strains and was verified by the population analysis profile/area under the curve (PAP‐AUC) method (Silveira *et al*. 2019). This study was conducted to observe whether the cell wall of the hVISA strain was also thickened using transmission electron microscopy (TEM), and preliminarily explore the molecular mechanism underlying the cell wall thickening of the hVISA strain by Real‐time quantitative PCR (RT‐qPCR).

## Results and discussion

### Cell wall thickness

**FIGURE 1:**
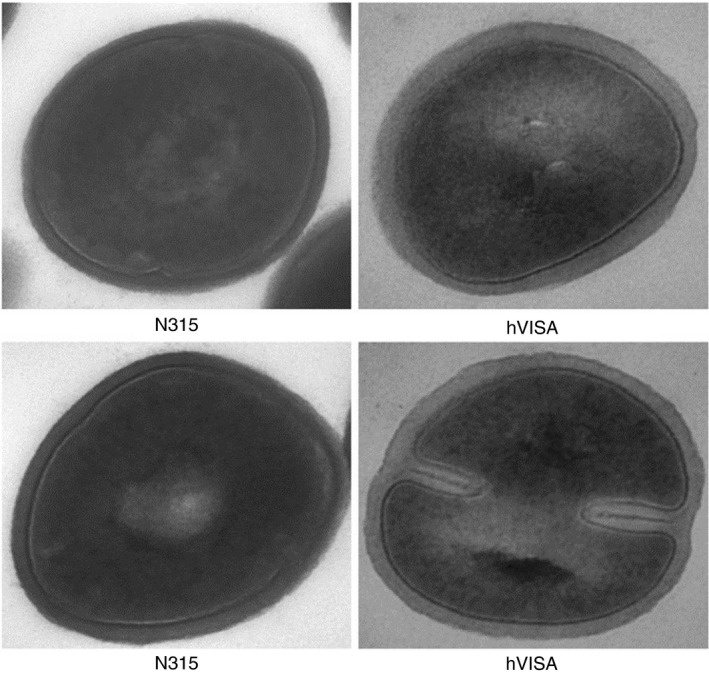
The cell wall images of N315 and hVISA under transmission electron microscope (×60 000 times magnification). The cell wall of the hVISA strain was thicker than that of the N315 strain (36·72 ± 1·04 nm vs 28·15 ± 1·25 nm, *P* < 0·05).

hVISA is rare in the clinic and the mechanism underlying the reduced sensitivity to VAN of this strain has not been elucidated (Bongiorno *et al*. 2020). The hVISA strain investigated in this study was derived from a patient in our hospital and the patient who had not exposed to VAN before. It may be helpful to study the mechanism underlying the reduced sensitivity to VAN exhibited by hVISA strains to prevent the development of further resistance to VAN. As shown in Fig. 1, the image captured by TEM showed that the cell wall of the hVISA strain was significantly thicker than that of the N315 strain (36·72 ± 1·04 nm vs 28·15 ± 1·25 nm, *P* < 0·05), although both strains were MRSA. Therefore, the morphology of the thickened cell wall supported that the reduced sensitivity to VAN exhibited by the hVISA stain might be attributable to the thickened cell wall.

### RT‐qPCR results of cell‐wall‐related genes

Taking the relative expression level of *glmS* gene as an example, the Ct values of the three RT‐qPCR assays are shown in Table S1. The results of 2^−ΔΔCt^ values between hVISA and N315 were 4·26:1, 3·81:1 and 3·92:1, and the two‐tailed Student’s *t* test showed that the expression of *glm0S* gene in the hVISA strain was significantly upregulated compared to the MRSA quality control strain N315 (*P* < 0·05). The RT‐qPCR results of other cell‐wall‐related genes were similar to those of *glmS* gene. The relative expression levels and significant differences of all cell‐wall‐related genes in the two groups are summarized in Table 1. The results showed that compared with the ordinary MRSA quality control strain N315, the expression of all cell‐wall‐related genes (*glmS*, *vraR*, *vraS*, *dlt*, *sgtB*, *murZ* and *PBP4*) in hVISA strains was significantly upregulated (*P* < 0·05).

**Table 1:** Relative expression levels of all cell‐wall‐related target genes and their statistical differences between hVISA and N315 strains

Target genes	2^−ΔΔCt^ (hVISA vs N315)			Student’s *t* test (*P*)
	First RT‐qPCR	Second RT‐qPCR	Third RT‐qPCR	
*glmS*	4·26:1	3·81:1	3·92:1	<0·05
*vraR*	2·53:1	2·39:1	2·17:1	<0·05
*vraS*	6·41:1	5·69:1	6·36:1	<0·05
*sgtB*	2·77:1	2·96:1	3·27:1	<0·05
*murZ*	4·19:1	3·22:1	3·58:1	<0·05
*PBP4*	3·55:1	3·31:1	3·53:1	<0·05

The results described above showed that the hVISA strain had significantly higher expression of cell‐wall synthesis‐related genes than N315 strains. And combing with the thickened cell wall, the higher expression of cell‐wall synthesis‐related genes further suggested that cell wall thickening might be the molecular mechanism underlying the reduced sensitivity to VAN of the hVISA strain. And the mechanism was consistent with the VAN resistance mechanism of the VISA strain (Mu50) (Cui *et al*. 2000) and VRSA strains (Cui *et al*. 2003).

l‐glutamine d‐fructose‐6‐phosphate aminotransferase (*glmS*) is one of the key enzymes in the cell wall peptidoglycan synthesis pathway (Matzner *et al*. 2017). Some researchers believed that the VISA strain could use glucose in the microenvironment to increase cell wall synthesis and bind more VAN to cell wall, preventing VAN from passing through the cell wall and thus inhibiting the antibacterial activity of VAN (Cui *et al*. 2000). The *glmS* gene expression of the clinically isolated hVISA strain in this study was almost four times higher than that of the N315 strain. The result indicated that the hVISA strain might increase the activity of *glmS* gene to use more glucose to synthesize more peptidoglycans, which, in turn, promotes cell wall thickening.

VAN‐resistance‐associated sensor/regulator (*vraS/R*) is a phosphotransferase‐mediated signal regulation system (Baseri *et al*. 2021). It is composed of histidine kinase *vraS* and response regulatory protein *vraR*. It can sense cell wall damage caused by various factors and respond quickly to fix the damage, so it is considered as cell wall stimulator (Dai *et al*. 2017). *vraS/R* plays an important role in low‐level VAN resistance (Gao *et al*. 2019), and some studies found that the expression of *vraS/R* was upregulated in the first VISA strain (named Mu50), the first hVISA strain (named Mu3) and the VISA strain (named JH9) compared to ordinary MRSA strain (Kuroda *et al*. 2000; McAleese *et al*. 2006). The *vraS/R* system involves many genes related to cell wall synthesis, including UDP‐*N*‐acetylglucosamine enolpyruvyl transferase (*murZ*) and monofunctional glycosyltransferase (*sgtB*) (Kuroda *et al*. 2003). *murZ* and *sgtB* are involved in the synthesis of cell wall monomer (Karinou *et al*. 2019; Boulhissa *et al*. 2020). The cell wall synthesis of *S. aureus* is a complex process, and the expression of various related genes is coordinated and inseparable. The *vraS/R* gene, *murZ* and *sgtB* were all upregulated in the hVISA in our study. It is inferred that the upregulation of the activity of these genes might be the molecular mechanism underlying the thickened cell wall and reduced sensitivity to VAN of hVISA.

Although the upregulated expression of the above‐mentioned cell‐wall‐related genes may be the molecular mechanism underlying the thickened cell wall of this hVISA strain, we observed that penicillin binding proteins (PBPs) were also upregulated, and this result was differed from the findings of previous reports (Sieradzki and Tomasz 2003; Navratna *et al*. 2010). PBPs are carboxypeptidase and transpeptidase and are mainly involved in the synthesis of the murein of the cell wall and the secondary cross‐linking of peptidoglycan, which play an important role in the growth and reproduction of bacteria and the maintenance of normal morphology (Miyachiro *et al*. 2019). A previous study suggested that after the *PBP4* gene of *S. aureus* was knocked out, the cross‐linking of peptidoglycan decreased significantly, VAN binding sites decreased and resistance to VAN increased (Navratna *et al*. 2010). Sieradzki and Tomasz (2003) also found that the VISA strains ‘JH9’ and ‘JH14’ both produced abnormally thickened cell walls with reduced peptidoglycan cross‐linking and significantly reduced *PBP4* expression levels. However, the expression of *PBP4* gene was upregulated in the hVISA strain, investigated in this study, which was also less sensitive to VAN. We surmise that at an earlier stage of VISA, a slight increase in the *PBP4* activity of hVISA can increase peptidoglycan cross‐linking, which can slightly increase the sensitivity of VAN. However, this effect cannot offset the reduced sensitivity to VAN caused by the upregulation of other genes related to the thickening of the cell wall. However, this report only presented our preliminary speculation, and the reason for the increased expression of *PBP4* gene of hVISA warrants further experimental research.

In conclusion, we confirmed that the cell wall of the hVISA strain was significantly thicker than that of ordinary MRSA strains. The upregulated expression of *glmS*, *vraR*/S, *sgtB*, *murZ* and *PBP4* genes related to cell wall synthesis might be the molecular mechanism underlying the cell wall thickening of the hVISA strain and might be related to its reduced sensitivity to VAN. Additionally, the limitation in this study needed to be clearly stated was that more hVISA strains might be needed to be tested to verify our hypothesis and conclusions, but we only tested one hVISA strain since hVISA was still a rare strain currently. However, with the continuous increase in hVISA and related researches, we believe that the results of our study could be used as a supplement to other hVISA‐related studies in the world and might be beneficial to prevent and control the emergence of more hVISA.

## Materials and methods

### Source of strains and test materials

The strains in this study included an hVISA strain and a VAN‐sensitive MRSA strain (the MRSA quality control strain named N315). The N315 strain was provided by the department of Clinical Microbiology in CPLAGH hospital. Just as mentioned in the Introduction, the hVISA strain was isolated from 130 clinical MRSA strains. In our previous study from 2013 to 2018, the 130 MRSA strains were sequentially isolated from patients' various specimens, such as sputum, blood, joint fluid, wound secretions, alveolar lavage fluid, ascites and so on. All the 130 strains were identified by the *S. aureus* latex agglutination test (Mérieux, Lyon, France), and their MICs of cefoxitin determined by the agar dilution method were all ≥8 *μ*g ml^−1^. The hVISA screening and confirmation were done using these 130 MRSA strains, which were sequentially screened by Brain Heart Infusion Agar Screening Method (46 positive strains out of the 130 MRSA strains), Macrodilution Etests (20 positive strains out of the 46 MRSA strains above) and PAP‐AUC method (only 1 positive strain out of the 20 MRSA strains above). The only one positive MRSA strain was confirmed as hVISA strain and preserved in −80°C refrigerator. When we conduct this study, the hVISA strain and the N315 strain were recovered from the refrigerator and cultured on blood agar, and then used in this study. Because this hVISA strain was just the MRSA strain that has been previously verified, no biochemical test was re‐performed in this study.

The main reagent materials in this study included bacterial culture medium (Oxoid Columbia Blood Agar plate, Thermo Fisher Scientific, UK), a total RNA extraction kit (RNeasy Mini Kit; Qiagen, Beijing, China), reverse transcription kit and One‐Step RT‐qPCR SYBR fluorescent dye (Dalian Bao Biological Engineering, Dalian, China) and an RT‐qPCR instrument (Applied Biosystems, ABI, UK). Total RNA extraction of MRSA bacteria and bacterial culture were implemented in a biological safety cabinet. Bacterial culture and RT‐qPCR were performed in our respiratory laboratory. Imaging of the bacterial cell wall by TEM was performed in the State Key Laboratory of Nephrology in our hospital.

### Bacterial culture

The hVISA and N315 bacteria were extracted from the −80°C medical refrigerator and thawed, and they were subsequently smeared and inoculated onto the blood plates by the traditional continuous streaking method. The blood plates were placed in a 37°C incubator for 24 h. The formation of independent isolated colonies for subsequent testing was the purpose of bacterial culture.

### Observation of the cell wall thickness of strains by TEM

The procedure to observe the cell wall thickness of strains by TEM was as follows: First, a single pure colony of hVISA and N315 grown on a blood plate was picked, washed with 1 ml of a 0·2 mol l^−1^ PBS solution (pH 7·4) and centrifuged at 8000 rev min^−1^ for 1 min, and the supernatant was discarded. Second, the strains were fixed with 2·5% glutaraldehyde for 1 h, fixed with 1% thorium tetroxide for 1 h, dehydrated with 30% ethanol for 15 min, dehydrated with 50% ethanol for 15 min, embedded in epoxy resin, ultrathin‐sectioned and double‐stained with uranium acetate/lead citrate. Finally, the images of the two strains were observed using TEM at 60 000× magnification. Upon randomly selecting 10 parts of the cell wall of each strain, the thicknesses of the 10 parts were measured and the average was calculated.

### Expression of cell wall thickness related genes of hVISA and N315

For extraction and reverse transcription of total RNA, an RNA extraction kit (RNeasy Mini Kit; Qiagen) was used to extract the total RNA. After extraction, the total RNA was quantified to ensure that the starting amount of mRNA in the reverse transcription system of all samples was equal. One‐step RT‐qPCR was performed in triplicate to detect the Ct (cycle threshold) values of the internal reference gene and the target genes. The relative quantification of the target gene (g*lmS*, *vraR*, *vraS*, *dlt*, *sgtB*, *murZ* and *PBP4*) to that of internal reference gene (16SrRNA) was used to evaluate the expression of each gene in the two strains using a 2^−ΔΔCT^ method, where ΔΔCt = (Ct value of the target gene of hVISA ‘minus’ Ct value of the internal reference gene of hVISA) minus (Ct value of the target gene of N315 ‘minus’ Ct value of the internal reference gene of N315). All the above experiments were performed according to the manufacturer’s instructions. All primer sequences applied in RT‐qPCR for the genes related to the cell wall synthesis of *S. aureus* are shown in Table 2, and all primer sequences were designed based on the sequence of each gene of MRSA N315 included in the Genebank. Primer design principles: (i) primer length is 18–27 bp; (ii) G + C content is 45–55%; (iii) *T*_m_ value is between 55 and 62°C and the *T*_m_ value difference between upstream and downstream primers does not exceed 2°C and (iv) RT‐qPCR product length should be less than 250 bp, and if the length was too long, the appearance of Ct value will delay and the RT‐qPCR reaction efficiency will be reduced since the RT‐qPCR reaction components might be excessively consumed.

**Table 2:** Primer sequences of cell‐wall‐related target genes and internal reference gene

Gene	Upstream primer	Downstream primer	Sequence length (bp)
Internal reference gene			
16SrRNA	GGCAAGCGTTATCCGGAATT	GTTTCCAATGACCCTCCACG	94
Cell wall related target genes			
*glmS*	CAATGTTACAAGTGACAAGC	CATTACTGCTGGTTGTTCAT	206
*vraR*	GTTATCTATCAACGCAAAG	TGTCTTCCATAAGTAAATCC	124
*vraS*	TTTATACCATACACTCGTACC	TTGCTGACTAACAGAATCG	169
*sgtB*	GCGATAATGTGGATGAACTAAG	ATTGTAGAATCGTTCATCTTCC	119
*murZ*	CGAAAAACAGTGCTGTAGC	CAGCATTTTGTATTTCAGTTGT	183
*PBP4*	GAGTTTGCCTGGTACAGAT	CTTACCATTTATCCTTTGC	244

### Statistical analysis

All statistical analyses were performed using spss version 22.0 software (SPSS Inc., Chicago, IL). The cell wall thickness was described as the mean (standard division, SD) or median (interquartile range, IQR), as appropriate according to normality. The statistical differences in cell wall thickness and the 2^−ΔΔCt^ between the hVISA strain and the N315 strain were analysed using a two‐tailed Student’s *t* test, and all results with a two‐tailed *P* < 0·05 were considered to be significant.

## Conflict of Interest

The authors have no conflicts of interest to declare. Additionally, this study was approved by the Clinical Trial Ethics Review Committee of the PLAGH Hospital, but formal consent was waived in our hospital.

## Author contributions

ZL designed the study and provided the experimental funding support. JC and HZ participated in the experiment and wrote this manuscript. ZM and MY participated in the experiment and revised this manuscript. All authors read and approved the submission of the final manuscript.

## Supporting information


**Table S1.** Relative expression levels of the *glmS* genes and their statistical differences between hVISA and N315 strains.
