# Supplementary material for: Cell wall thickness and the molecular mechanism of heterogeneous vancomycin‐intermediate Staphylococcus aureus
Source: Lett Appl Microbiol. 2021 Feb 15;72(5):604–9. doi: 10.1111/lam.13456 (PMC8248079; doi:10.1111/lam.13456)
Supplement: Supplementary file 1 — Table S1. Relative expression levels of the glmS genes and their statistical differences between hVISA and N315 strains. [file LAM-72-604-s001.docx]

**Supporting information**

| **Supplementary Table. Relative expression levels of the *glmS* genes and their statistical differences between hVISA and N315 strains.** | | | | | | | |
| --- | --- | --- | --- | --- | --- | --- | --- |
|  | **N315 (Ct)** | | **hVISA (Ct)** | |  | **hVISA vs N315** | |
|  | *glmS* | 16SrRNA | *glmS* | 16SrRNA |  | 2^-ΔΔCt^ | Student’s t-test |
| First RT-qPCR | 25.56 | 19.58 | 22.79 | 18.90 |  | 4.26:1 | P<0.05 |
| Second RT-qPCR | 25.46 | 19.57 | 22.65 | 18.69 |  | 3.81:1 |  |
| Third RT-qPCR | 25.51 | 19.66 | 22.72 | 18.84 |  | 3.92:1 |  |
| Ct, Cycle threshold; ΔΔCt= (Ct value of *glmS* of hVISA “minus” Ct value of 16SrRNA of hVISA) minus (Ct value of *glmS* of N315 “minus” Ct value of 16SrRNA of N315). | | | | | | | |
